# Dengue Vaccines: A Perspective from the Point of View of Intellectual Property

**DOI:** 10.3390/ijerph120809454

**Published:** 2015-08-12

**Authors:** Claudimar Pereira da Veiga, Cássia Rita Pereira da Veiga, Jansen Maia Del Corso, Wesley Vieira da Silva

**Affiliations:** Pontifical Catholic University of Paraná, PUCPR, Rua Imaculada Conceição, 1155 Prado Velho, 80215-901 Curitiba, PR, Brazil; E-Mails: cassia.veig@gmail.com (C.R.P.V.); del.corso@pucpr.br (J.M.D.C.); wesley.vieira@pucpr.br (W.V.S.)

**Keywords:** viruses, heath public, innovation, patent, pharmaceutical industry, dengue virus, neglected tropical disease

## Abstract

Dengue is a serious infectious disease and a growing public health problem in many tropical and sub-tropical countries. To control this neglected tropical disease (NTD), vaccines are likely to be the most cost-effective solution. This study analyzed dengue vaccines from both a historical and longitudinal perspective by using patent data, evaluating the geographic and time coverage of innovations, the primary patent holders, the network of cooperation and partnership for vaccine research and development (R & D), the flow of knowledge and the technological domain involved. This study can be seen as an example of the use of patent information to inform policy discussions, strategic research planning, and technology transfer. The results show that 93% of patents were granted since 2000, the majority belonging to the United States and Europe, although the share of patents from developing countries has increased. Unlike another NTDs, there is great participation of private companies in R & D of dengue vaccines and partnerships and collaboration between public and private companies. Finally, in this study, the main holders showed high knowledge absorption and generated capabilities. Therefore, this issue suggests that to overcome the difficulty of translational R & D it is necessary to stimulate the generation of knowledge and relevant scientific research, to enable the productive sector to have the capacity to absorb knowledge, to turn it into innovation, and to articulate partnerships and collaboration.

## 1. Introduction

Dengue is a serious infectious disease caused by a virus, which is transmitted by Aedes mosquitoes. Since the first isolation of dengue virus (DENV) in 1943, four phylogenetically and antigenically distinct dengue viruses have been identified (DENV 1–4) [1]. Dengue has evolved from a sporadic disease to a major public health problem with substantial social and economic effects because of the increased geographical extension, the number of cases, and the disease severity [2,3,4]. There are many factors that have contributed to this emergency of epidemic dengue, but three of them have been the principal drivers: (i) urbanization and changed environmental conditions [5]; (ii) globalization [6]; and (iii) breakdown in public health infrastructure and vector control programs [2]. Dengue is a neglected tropical disease (NTD), but it belongs to a distinct category due to its acute symptoms and higher mortality rate [7]. Its transmission is ubiquitous throughout the tropics, with the highest risk zones in the Americas and Asia [8]. It is estimated that there are 390 million dengue infections per year in the world, including 96 million clinically symptomatic cases [8]. The total annual mortality is estimated to be about 22,000 cases [9].

Dengue is a growing public health problem in many tropical and sub-tropical countries, because vector control has proved to be of limited effectiveness and there are no available dengue specific antiviral therapies. Novartis Institute of Tropical Disease, a private-public fund that has made significant contributions to dengue biology and drug discovery, was founded in 2002 [10]. Although the effort has not yet led to the development of a clinical candidate, the experience accumulated during the past decade has provided a better rationale for the on-going anti-DENV effort. On the other hand, antigens’ discovery and pre-clinical development of dengue vaccine have progressed much beyond early development, with six candidates currently in clinical trials [11]. These vaccines employ a variety of approaches, including molecularly defined live-attenuated viruses, inactivated viruses, recombinant subunits, and DNA (DVI, 2012). Overall, the use of vaccines as the solution with the best cost-benefit-effectiveness for NTD has been suggested, either in isolation or in combination with specific drugs as part of a global control program [12,13].

A lot of empirical studies have evaluated dengue vaccines [9,11,14,15], but the topic was explored only once from the point of view of Intellectual Property (IP) [16]; this study reviewed the scientific basis and status of the dengue vaccines under development, identified key players, and licensing status of limited patents. Conversely, this study analyzes dengue vaccines from both historic and longitudinal perspectives and uses different methodologies and databases. Thus, using patent data, this study aims to analyze the geographic and time coverage of innovations, the main patent holders and the network of cooperation and partnership for vaccine R & D, and finally, the flow of knowledge and technological domains involved. It is fundamental to answer some questions from the point of view of IP: (i) When did dengue vaccine inventions experience accelerated development? (ii) Which countries/institutions are involved in dengue vaccine inventions? (iii) Which institutions have joint propriety of their dengue vaccine inventions (co-ownership)? (iv) Which companies can be considered high adoption of knowledge and which have the ability to generate and export innovative outputs? (v) What is the technological domain of dengue vaccine inventions? This study can be seen as an example of using patent information to understand strategic planning of the pharmaceutical industry. Patents data are science and technology indicators [17] and patent findings can provide insights that cannot be captured using other sources and contribute, among other things, to inform policy discussions, strategic research planning, and technology transfer [16,18]. This study is divided into four parts, including this introduction. Section 2 describes the methodology, Section 3 discloses the results, and Section 4 presents the final considerations.

## 2. Materials and Methods

**Figure 1:**
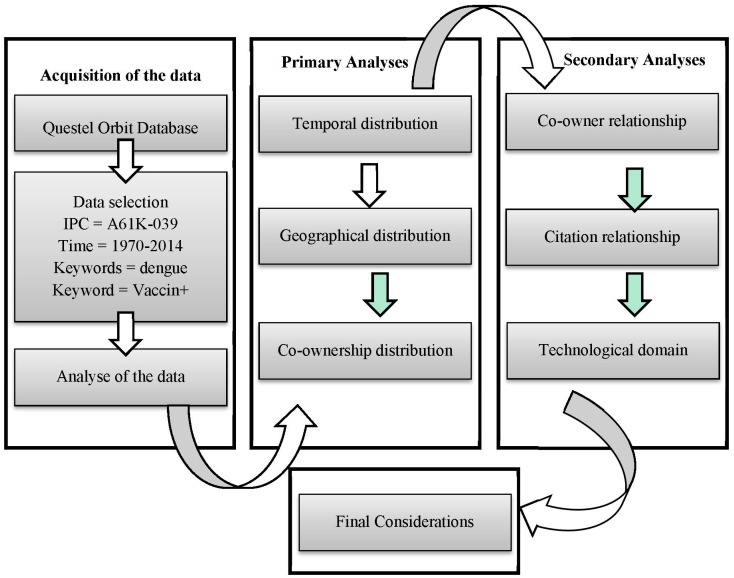
Type, acquisition and treatment of the data under study.

This study used data from patent families published between 1970 and 2014 that were retrieved from Questel Orbit^®^ (Sao Paulo, Brazil) a patent database with integrated Web based patent analysis software. Unlike other patent databases, Questel Orbit^®^ allows patent analysis visualizations, designed to give answers to key business questions. Data were selected using the International Patent Classification (IPC) code A61K-039, which refers to medicinal preparations that contain antigens and antibodies. The search was refined using the keywords “dengue” and “vaccin+” in the title and abstract in order to differentiate patent documents related to another vaccines and diagnostic methods. The command line was ((DENGUE)/TI/AB AND (VACCIN+)/TI/AB) AND (A61K-039)/IPC. All patent data from the database were primary analyzed to establish temporal distribution, geographical distribution and co-ownership distribution (see Figure 1). In a second moment, patent data were analyzed about co-owner relationships, citation relationships and technological domains (see Figure 1). The search results were correlated with other data sources to provide insights not obtained by other studies.

## 3. Analysis and Results

### 3.1. Temporal and Geographical Distribution of Patent Families

**Figure 2:**
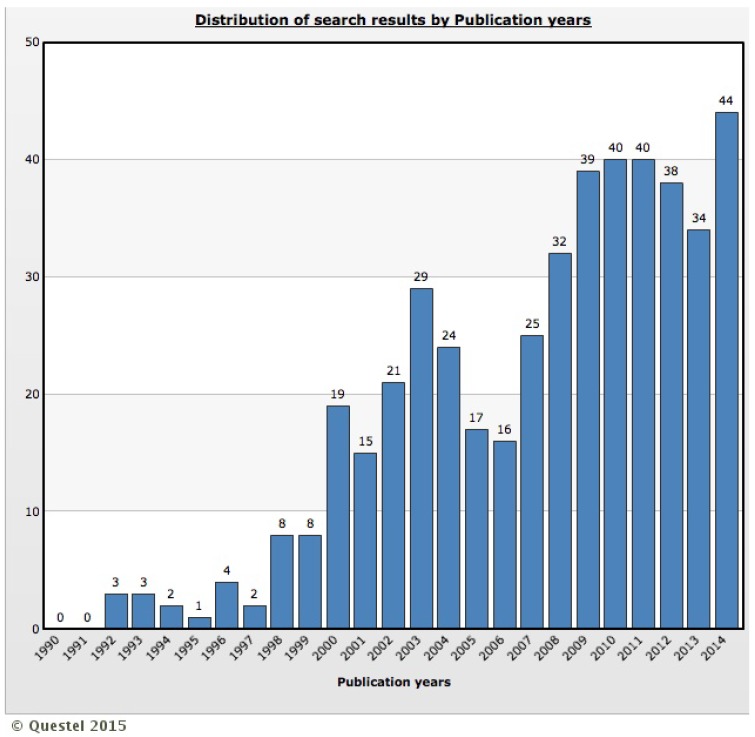
Distribution of number of published patents by publication year from 1990 to 2014.

In total, the selection used in Questel Orbit^®^ database classified 151 patent families during the period from 1970 to 2014. Considering the year of first application to analyze the patent families, 13 documents were subjected before 1996, 25 documents between 1996 and 2000, 24 documents between 2001 and 2005, 47 documents between 2006 and 2010 and 42 documents after 2010, which demonstrates a growing interest in innovations related to dengue vaccines throughout the years. This is confirmed by Figure 2, which illustrates the distribution of number of published patents by publication year. Although the analysis starts in 1970, Figure 2 illustrates the results only since 1990 because between 1970 and 1989 only three patents were granted. The Figure 2 illustrates that 433 patents (93% of the patents) were granted since 2000, which coincides with the period of highest incidence and social and economic concerns caused by the disease. Epidemiologically in the last 10 years there has been a rapid and marked increase in the number of reported dengue outbreaks in countries with tropical and subtropical climates [9].

**Figure 3:**
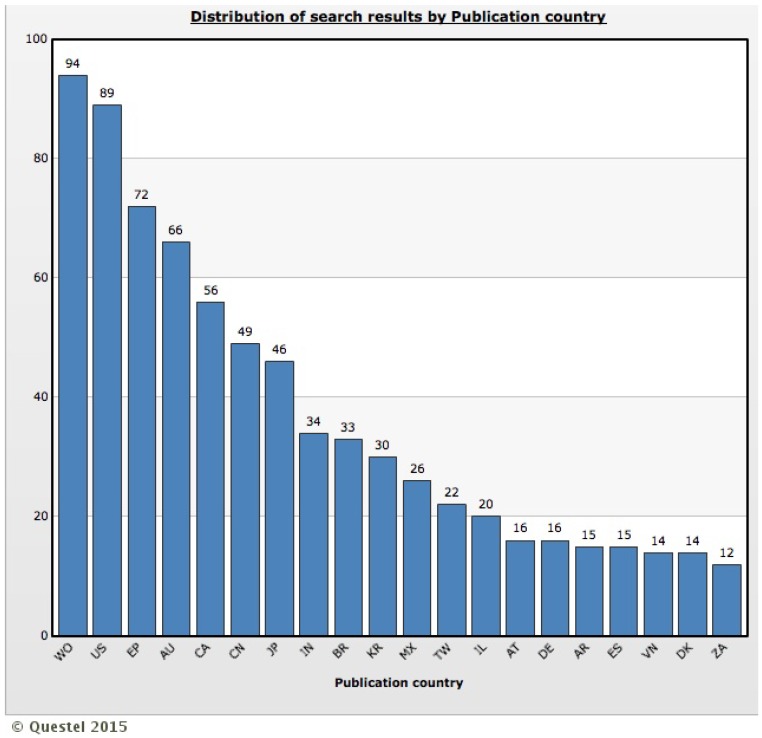
Distribution of number of published patents by top 20 publication countries. WO: World Intellectual Property Organization, US: United States of America, EP: European Patent Office, AU: Australia, CA: Canada, CN: China, JP: Japan, IN: India, BR: Brazil, KR: Korea (South), MX: Mexico, TW: Taiwan, IL: Israel, AT: Austria, DE: Germany, AR: Argentina, ES: Spain, VN: Viet Nam, DK: Denmark, ZA: South Africa.

Figure 3 presents the distribution of number of published patents by top 20 publication countries. The large number of patents granted by the World Intellectual Property Organization (WIPO) demonstrates high interest, on the part of the patent holders, for filing patent applications to protect inventions in each of the WIPO contracting states. In Figure 3, it is also possible to note a large number of patents granted to the United States of America (US) and Europe (EP, AT, DE, ES, DK), which may be related to the nationality of depositors, often belonging to these countries [19], or interest in the pharmaceutical market in that region. It is important to remember that the US and Europe are among the top 10 pharmaceutical markets in the world [20] and, in addition, during the last decade the local dengue transmission has also been documented in parts of these countries [21,22,23].

Finally, as documented in previous studies [16], only a few patent applications have been filed in developing countries. The countries in which more than 30 patents have been filed include China, India, Brazil and Republic of Korea. Brazil and India, two innovative developing countries [24] have dengue vaccines in early development stages [25]. Specifically, the increasing co-circulation of DENV in most regions of the world, particularly in Australia, Asia and Latin America, has important implications for patterns in disease severity and hyperendemicity, as well as for ongoing vaccine efforts designed for these markets [1]. Over time, however, one would expect a trend towards greater patent application filings in developing countries, but past activity should not be construed as a reliable predictor of future activities because a number of patent applications may still enter the national phase under PCT (Patent Cooperation Treaty) filing in developing countries [16].

**Figure 4:**
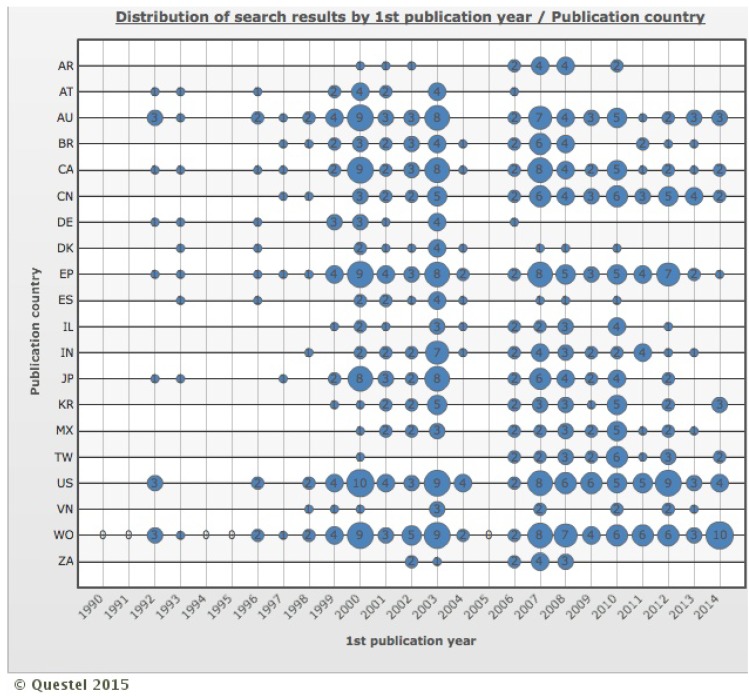
Distribution of search results by correlation between first publication year and top 20 publication countries from 1990 to 2014. AR: Argentina, AT: Austria, AU: Australia, BR: Brazil, CA: Canada, CN: China, DE: Germany, DK: Denmark, EP: European Patent Office, ES: Spain, IL: Israel, IN: India, JP: Japan, KR: Korea (South), MX: Mexico, TW: Taiwan, US: United States of America, VN: Viet Nam, WO: World Intellectual Property Organization, ZA: South Africa.

Figure 4 presents the distribution of search results by correlation between the first publication year and the top 20 publication countries. Only six patent offices have granted patents every year in the last nine years (AU, CA, CN, EP, US and WO). Two patent offices did not grant any patent since 2006 (AT and DE), one since 2008 (ZA) and other two since 2010 (DK and ES).

In Figure 4, it is possible to note two distinct periods of intense publication of patents for the top 20 publication countries, 2000 to 2003 and 2007 to 2010. Core patents of key players suggest that the first period is related to DNA, reverse genetically and chimeric vaccines and the second period with chimeric vaccines again as well as recombinant virus protein vaccines [16]. The knowledge accumulated during the past decades was fundamental to the granted patents in the last decade.

### 3.2. Holders and Co-Ownership Distribution of Patent Families

Table 1 illustrates the distribution of search results by main holders according to the number of patents. Importantly, under the name “US Department of Health & Human Services” patents were added to various institutes attached to the US Government such as “US Navy”, “US Army”, “US Government”, and “US National Institutes of Health”. From 74 holders listed in Table 1, half refer to public institutions and of these 16 are universities. Most of the NTDs vaccines are currently being developed in the nonprofit sector [26], but dengue is an important exception. Most often, nonprofit sector is unable to overcome the barriers that exist to transform research results into products and bring them to the market. In the case of dengue vaccines, the strong participation of industry ensures financial resources required for the licensing, manufacturing, and global access to vaccines [26,27].

**Table 1:** Distribution of search results by main holders.

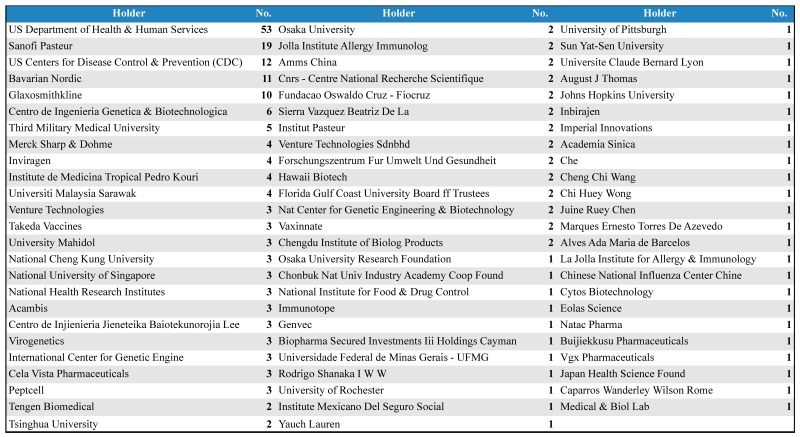

Many studies cite the importance of public-private partnership as an essential factor for the development of solutions for NTD [7,12,13]. It is hard to measure this partnership in IP, but the co-ownership innovations tool is a powerful instrument for analyzing scientific and technological collaborations and partnerships. However, it is important to remember that the pharmaceutical industry is slowly absorbing the idea of collaborative patent license models through both patent pool and clearinghouse [28] and this relationship is not identified by the co-ownership tool of Questel Orbit^®^ database.

**Figure 5:**
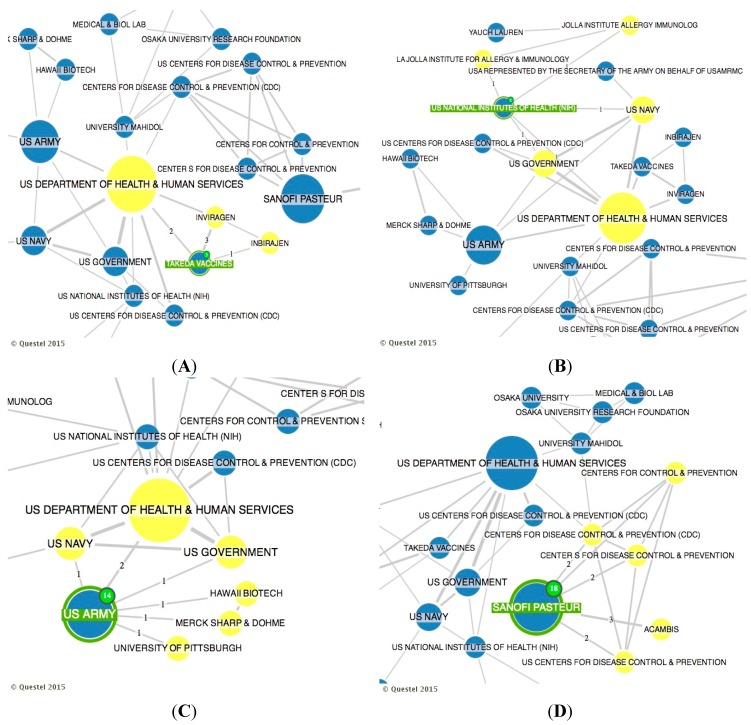
Distribution of search results by main co-ownerships of late-stage dengue vaccine developers. Part **A**: co-ownership of Takeda; Part **B**: co-ownership of US National Institute of Health; Part **C**: co-ownership of US Army; Part **D**: co-ownership of Sanofi Pasteur.

Figure 5 presents the distribution of search results by co-ownership only of late-stage dengue vaccine developers [25]. Network node sizes are displayed in proportion to their “convergence points”, an indicator of the gatekeeper/broker role of the node in the network. In Figure 5 (Part A), it is possible to examine the relationship between the two private pharmaceutical companies Takeda and Inviragen. These private companies jointly announced in 2013 that they have entered into a definitive agreement for Takeda to acquire Inviragen. This acquisition combines Inviragen’s expertise in both viral vaccine P & D and extensive worldwide network of preclinical and clinical collaborators with Takeda’s resources, product development expertise, and global reach [29]. Figure 5 (Part A) also illustrates that Takeda has worked with the US Government and its product, a live attenuated tetravalent recombinant chimeric vaccine candidate using an attenuated DENV-2 backbone, is currently in phase II clinical development [25].

Figure 5 (Part B) demonstrates the co-ownership of US National Institute of Health (NIH). It is possible to look at the relationship of various US government agencies and the relationship of NIH with Jolla Institute for Allergy & Immunology, an international leader of biomedical research founded by the US Government. The NIH has become the first patent-holder to share its IP with the Medicines Patent Pool, an initiative newly-established with the support of UNITAID [28], an innovative global health financing mechanism which was co-founded by Brazil, Chile, France, Norway and the United Kingdom in 2006. Several industrial sponsors in Asia and Brazil have been awarded nonexclusive licenses for NIH [16] to commit in making the results of their research globally available. This is the case of the NIH-Butantan Foundation’s (Brazil) live attenuated tetravalent dengue vaccine that is currently in phase II clinical trial [25], but this relationship of patent licensing is not captured by the co-ownership tool of Questel Orbit^®^ database.

Figure 5 (Part C) illustrates the co-ownership of the US Army. Whole virus inactivated vaccines have been produced by Putnak and colleagues of the Walter Reed Army Institute of Research (WRAIR) [16], but the PI belongs to the US Army. This process is a collaborative development between WRAIR Antigen/GSK adjuvants vaccine and Oswaldo Cruz Foundation (Brazil). All three parties have contributed to preclinical and clinical R & D [30]. A tetravalent whole virus dengue purified inactivated vaccine is in early Phase I trials in the continental USA and in Puerto Rico [25]. US Army also has co-ownership with both Merck Sharp & Dohme and Hawaii Biotech and partnerships with several US Government agencies, but these co-ownerships did not produce late-stage dengue vaccines [25]. It is important to highlight the wide participation and co-ownership of US government institutions in R & D of dengue vaccines. Besides dengue being a serious infectious disease, government institutions have a unique interest in viral vaccines due to bioterrorism that involves deliberate release of viruses, bacteria, or their toxins to cause morbidity and mortality in humans [31]. Dengue belongs to the class of third highest potential bioterrorism agents, which includes emerging pathogens that could be engineered for mass dissemination in the future because of availability, ease of production and dissemination [32].

Figure 5 (Part D) demonstrates the co-ownership of Sanofi Pasteur, which has completed clinical testing with a live attenuated tetravalent recombinant chimeric vaccine using a yellow fever backbone [15]. The original owner of necessary technologies was St. Louis University, which granted an exclusive license to Acambis, which was then exclusively sub-licensed to Sanofi Pasteur along with the entire package of Acambis patents. Moreover, Center for Disease Control and Prevent (CDC) and colleagues have developed a dengue-dengue homologous chimera through a collaborative effort involving Sanofi Pasteur [16]. These relationships are illustrated in Figure 5 (Part D). In general, one can say that late-stage dengue vaccine developers are intense co-owners of public–private institutions and, in most cases, there are large pharmaceutical companies involved.

**Figure 6:**
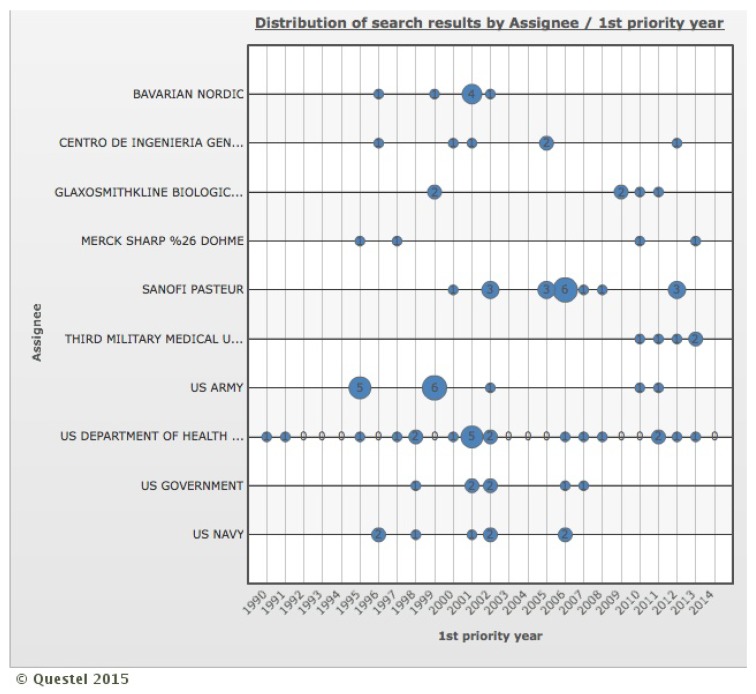
Distribution of search results by the correlation between first priority year and top 10 holders. Holders: Bavarian Nordic, Centro de Ingenieria Genetica Biotechnologica, Glaxosmithkline Biologicals, Merck Sharp & Dohme, Sanofi Pasteur, Third Military Medical University, US Army, US Department of Health & Human Service, US Government, US Navy.

Still, in regards to holders and co-ownership distribution of patent families, Figure 6 illustrates the distribution of search results by the correlation between the first priority year and the top 10 holders. It is possible to note that Bavarian Nordic, the fourth largest holder of the survey data, has made no priority publications since 2002. The US Navy and US Government are in a similar situation and have produced no priority publications since 2006 and 2007, respectively. On the other hand, Glaxosmithkline and Third Military Medical University concentrate their priority publications over the past five years while Sanofi Pasteur and US Department of Health & Human Service show a more uniform distribution of patents over time. These data do not allow making inferences about the continuity of R & D, conducting clinical trials or granting the IP right to a new holder.

### 3.3. Flow of Knowledge and Classification of Holders

The theoretical and empirical literature about innovation suggests that patent citations provide a way to evaluate the flow of knowledge from an original “stock” and, therefore, reveal the imitation and diffusion of a given idea [33]. Patent citations reveal the cooperation network for a given technology and the informal dialogue with exchanges of information among companies and countries [34,35]. Pieces of knowledge may originate from both the internal and external boundaries of the organization [36,37], and in this sense, companies can be classified as organizations that either generate innovations or that adopt them. Both types of organizations are innovative, albeit they follow different paths: for the first, the ability to generate innovative outputs is crucial, whereas the second develops the ability to absorb innovative inputs [38,39].

Organizations responsible for 151 patents in this study were classified according to their citation by “holders citations” tool of Questel Orbit^®^ (limitation of three patent families per holder, minimum of one citing patent family and hidden orphan nodes). The arrow directions indicate the citing documents, starting from those that were cited by them. The colors indicate the holders with citations for the selected node: green color indicates the company in question, red for cited companies, yellow for citing companies and orange for both citing and cited company in the same node. Companies with many citing patents were considered to have a high adoption of knowledge by the ability to absorb innovative inputs. Companies cited by other organizations were considered as high generation by their ability to generate and export innovative outputs. It is important to note that this analysis did not identify coincident organizations with different names of registry; as such, some important companies were not evaluated (such as Glaxosmithkline).

**Figure 7:**
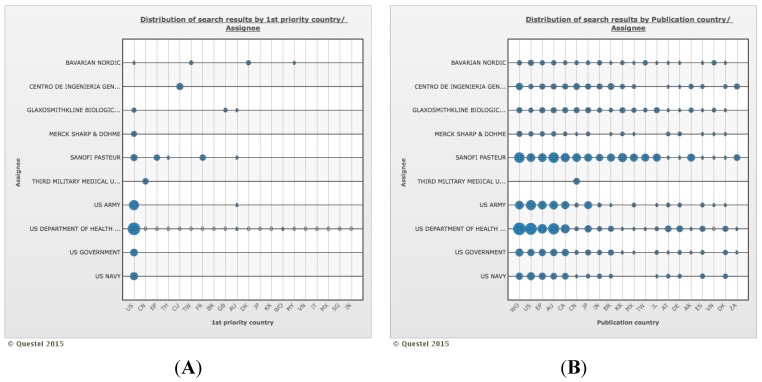
Distribution of search results by the correlation between top 10 holders and priority country (part **A**) and publication country (part **B**). Holders: Bavarian Nordic, Centro de Ingenieria Genetica Biotechnologica, Glaxosmithkline Biologicals, Merck Sharp & Dohme, Sanofi Pasteur, Third Military Medical University, US Army, US Department of Health & Human Service, US Government, US Navy. Countries: AR: Argentina, AT: Austria, AU: Australia, BR: Brazil, CA: Canada, CN: China, CU: Cuba, DE: Germany, DK: Denmark, EP: European Patent Office, ES: Spain, FR: France, GB: United Kingdom, IL: Israel, IN: India, IT: Italy, JP: Japan, KR: Korea (South), MX: Mexico, MY: Malaysia, SG: Singapore, TH: Thailand, TW: Taiwan, US: United States of America, VN: Viet Nam, WO: World Intellectual Property Organization, ZA: South Africa.

Figure 7 illustrates the distribution of search results by correlation between top 10 holders and both priority country (Part A) and publication country (Part B), respectively. Commonly, the priority publication (Part A) occurs in the country of holder’s origin or in the market of most interest for trade (mainly US). Moreover, most of the top 10 holders’ patents were filed in developed countries with only a small number also filed in selected developing countries such as China, India, Brazil, Republic of Korea, Mexico, Taiwan, Argentina, South Africa and Vietnam (Part B).

**Figure 8:**
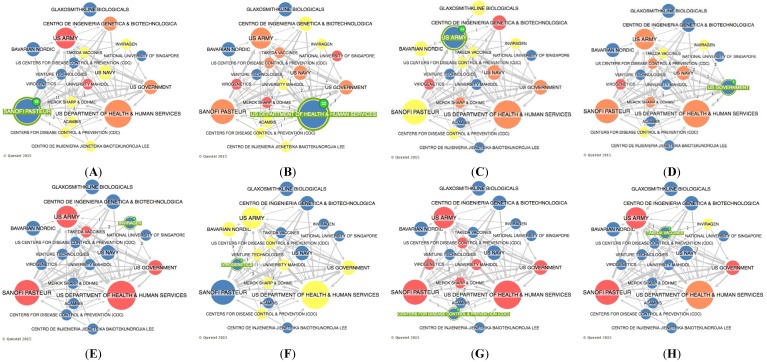

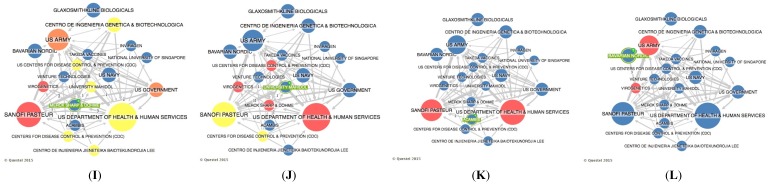
Holders’ citations of main companies/institutions. Flow of knowledge of Sanofi Pasteur (Part **A**), US Department of Health and Human Service (Part **B**), US Army (Part **C**), US Government (Part **D**), Inviragen (Part **E**), Virogenetics (Part **F**), Center for Disease Control and Prevent (Part **G**), Takeda (Part **H**), Merck Sharp & Dohme (Part **I**), University Mahidol (Part **J**), Acambis (Part **K**), Bavarian Nordic (Part **L**).

The following analysis refers to Figure 8. In general, Figure 8 illustrates the great flow of knowledge among all analyzed holders. All private companies have citing/cited patents with US Government agencies (Part A, H, I, L). It is observed also an intense flow of knowledge among several US government agencies (Part B, C and D) and perhaps this is the superior performance reason reached for this country in the number of filed patents for dengue vaccines. Some holders such as Inviragen (Part E), CDC (Part G), Acambis (Part K) and Bavarian Nordic (Part L) display exclusively generator capabilities, while virogenetics (Part F) presents a totally opposite result, in other words, exclusive absorber capabilities.

**Figure 9:**
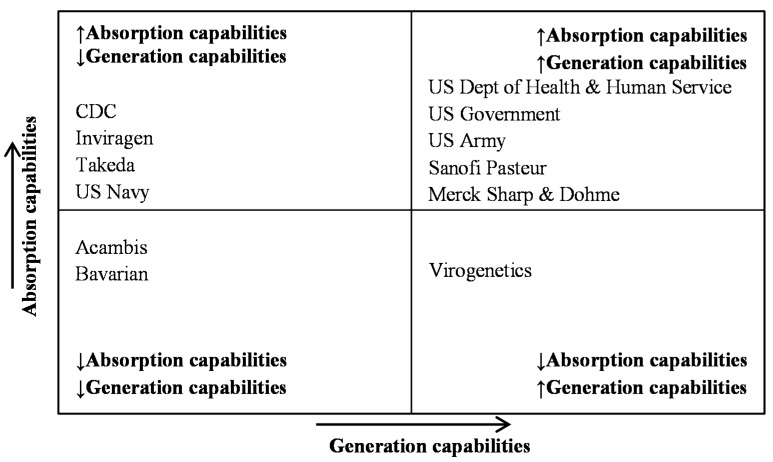
Holders’ classification by the number and type of citation.

Figure 8 also demonstrates the presence of a small number of universities involved in the flow of knowledge (only Mahidol—Part J and Virogenetics—Part F) which suggests some conclusions: (i) universities have generated few patents/R & D on the topic at hand; (ii) universities may not be responsible for the basic knowledge required for the technologies involved in this type of innovation; and (ii) universities can transfer the generated knowledge through other sources such as conferences and scientific papers. The analysis of Figure 8 and Figure 9 is also useful as an empirical basis for evaluating mergers and acquisitions in the pharmaceutical industry as these processes may occur due to the need to expand portfolios, with the goal of acquiring new technology [40] or the need for legal adjustments to the use of technology protected by IP rights [41]. It is important to remember that Inviragen was acquired by Takeda and the Acambis by Sanofi Pasteur. The assessment of both co-ownership and co-citation shows strong correlation between these companies (Figure 8, Parts A and H). Finally, the analysis of Figure 8 may also be of interest for evaluating the technological overlap of products in development since any company whose patents are citing a given patent is likely to be operating in the same technological area [34]. This complex analysis is beyond the scope of this work, but studies about the technological base of dengue vaccine suggest technical proximity of some products like those patented by Inviragen (Takeda), Acambis (Sanofi-Pasteur) and NIH, which have technologies that are all variants of the Chimeric Live Attenuated Dengue Vaccines [16]. The flow of knowledge established between these companies can be seen in Figure 8 (Part A). Finally, Figure 9 illustrates the classification of each holder by the number and type of citations. US Government agencies, Inviragen, Takeda, Mahidol University, Sanofi Pasteur and Merck Sharp & Dohme can be considered higher absorptive capability companies and US Government agencies (except CDC), Sanofi Pasteur, Merck Sharp & Dohme and Virogenetics have higher ability to generate and export innovative outputs. It is important to note that late-stage dengue vaccine developers [25] such as Takeda, US Army (Oswaldo Cruz Foundation), and Sanofi Pasteur have higher absorption capabilities. The main holders (US Department of Health and Human Service) showed both high absorption of knowledge and generation of capabilities.

### 3.4. Technological Domain

**Figure 10:**
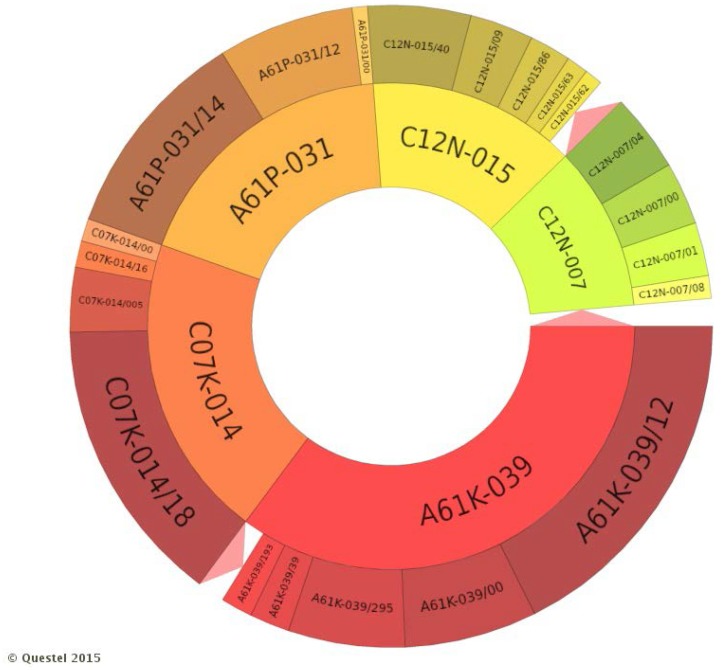
Technological fields of search results.

Figure 10 illustrates the most common IPC codes of search results. Obviously, the IPC used in the selection of patents, A61K-039, is the most common. This larger class has some important sub classifications related to viral antigens (A61K-039/12), polyvalent viral antigens (A61K-039/295) and formulations containing immune-stimulating additives (A61K-039/39). The second class related to human needs, A61P-031, refers to anti-infective, preferably antiviral (A61P-031/12) for RNA viruses (A61P-031/14). However, as the focus is to analyze the technological expertise of selected patents, the classification “C” which refers to chemical compounds may be more interesting.

The second most frequent class in Figure 10 is the C07K-014 which refers to peptides containing more than 20 amino acids and that is subdivided into DNA virus peptide such as Epstein-Barr virus (C07K-014/005), RNA virus peptide as HIV-1 (C07K-014/016), and RNA virus peptide of the family Togaviridae, such as flaviviruses for example (C07K-014/018). Following, the C12N-015 class depicts the processes related to mutation or genetic engineering, DNA or RNA concerning genetic engineering and preparing vectors which may occur by recombinant DNA technology (C12N-015/009), by preparing mutants without introduction of exogenous genetic material, by using DNA or RNA fragments or genes that encode animal protein/viral proteins/RNA virus protein such as Flavivirus (C12N-015/040), by using DNA sequences that encode fusion proteins (C12N-015/062) or by the introduction of exogenous genetic material using both regulation of the expression and viral (C12N-015/086) vectors (C12N-015/063). The less frequent class C12N-007 refers to the composition, preparation or purification of virus/bacteriophage (C12N-007/00), and the proceedings for inactivation or attenuation (C12N-007/04), including the one regarding the transfer virus in the series (C12N-007/08).

## 4. Final Considerations

This study analyzed dengue vaccines from both longitudinal and historical perspectives and answered some important questions from the point of view of intellectual property. It can be seen as an example of using patent information to inform policy discussions, strategic research planning, and technology transfer. Nowadays, dengue is regarded as the most prevalent and rapidly spreading viral disease in human beings [2] and it causes more human morbidity globally than any other disease transmitted by mosquitoes [11]. It is likely that during the next decade, dengue will expand its geographic reach and become an increasing burden on health resources in affected areas [11]. Certainly, the potential solution for this viral disease is not yet available and as there is a market in the private sector for vaccine development of DENVs, much effort to develop vaccines has been driven by private companies and government. The results demonstrate great participation of private companies in R & D of dengue vaccines and partnerships and collaboration between public and private companies. In the process of finding potential solutions for NTDs, there are few precedents to obtain licensing and global access to vaccines without the support of large pharmaceutical companies, either through collaboration in R & D or because these companies are holders of expertise and financial resources required for the licensing, manufacturing, and global access to vaccines [27]. In relation to public policy, it is important to highlight that the simple distribution of financial incentives is not an effective measure if the parties involved are not prepared to cooperate in an innovative environment [42]. In this study, the primary patent holders (US Department of Health and Human Service) showed both high knowledge absorption and generation of capabilities, and this issue suggests that to overcome the difficulty of translational R & D, it is necessary to stimulate the generation of knowledge and relevant scientific research, to enable the productive sector to have the capability to absorb knowledge and to turn it into innovation, and finally to articulate partnerships and collaboration. There are many NTDs in developing countries that require urgent medical attention, and reflections on these issues can be useful for policy makers.

Although there are still technical obstacles confronting the development of dengue vaccines, the introduction of this vaccine to the market appears to be imminent. It is important to consider that even if the attractiveness of multinationals increases, doubt remains whether the innovation achieved can deliver products with a better access and cost-benefit compared to the traditional model guided by the market. The vaccine introduction must first resolve more immediate problems of access to medicine and health system sustainability in less developed countries [13]. In addition, there are important questions that will need to be answered regarding the best system for incorporation of dengue vaccines [43] such as clear guidelines on the use of dengue vaccines, the best system to be adopted for the incorporation of vaccines into the National Immunization Program, and the challenges related to the distribution and demand forecast of new biological products [13]. Finally, it is necessary for the public sector to plan the introduction strategy and implementation of dengue vaccines to reduce the very lengthy time it has taken for vaccines to be introduced [44].

The findings presented herein and their interpretation should be considered within the context of the limitations of the study, primarily concerning the use of patent data. It is important to consider that not all inventions meet the patentability criteria [45]. In addition, the patented inventions may differ in “quality”—*i.e.*, in the magnitude of the inventive output associated with them [46]—and the protection of IP, which has increased worldwide since the 1990s, as has the probability of patent registration and quantity of citation. Despite these limitations, patent data should not be dismissed as a statistical indicator because they can provide information not captured by other sources, particularly if understood in an integrated manner and analyzed with a view to establish correlations with other data. Analyses of co-ownership and citations, for example, were useful for assessing trends in mergers and acquisitions, overlap of products, technological collaborations and partnerships. Other limitations of the present study were the selection of a specific pharmaceutical segment, temporal constraints, and the selection of patents using the IPC and keyword searches. The procedure adopted here can be applied to other selection criteria, in other pharmaceutical markets, in different industrial sectors, and in distinct periods. This subject is complex, and the present study did not intend to exhaustively address it.
